# 高剂量伏美替尼联合培美曲塞鞘内化疗治疗*EGFR*突变型非小细胞肺癌软脑膜转移的疗效与安全性分析

**DOI:** 10.3779/j.issn.1009-3419.2025.101.24

**Published:** 2025-12-20

**Authors:** Xin CHEN, Mingyang HE, Cen CHEN, Cheng JIANG, Huiying LI, Yongjuan LIN, Tingting YU, Yu XIE, Aibin GUO, Mingmin HUANG, Zhenyu YIN, Tianli ZHANG

**Affiliations:** ^1^210008 南京，南京中医药大学附属鼓楼医院老年科, 210008; ^1^Department of Geriatrics, Nanjing Drum Tower Hospital, Clinical College of Nanjing University of Chinese Medicine, Nanjing 210008, China; ^1^210008 南京，南京大学医学院附属鼓楼医院老年肿瘤科; ^2^Department of Geriatric Oncology, Nanjing Drum Tower Hospital, Affiliated Hospital of Medical School, Nanjing University, Nanjing 210008, China

**Keywords:** 肺肿瘤, 软脑膜转移, 表皮生长因子受体, 伏美替尼, 培美曲塞鞘内化疗, Lung neoplasms, Leptomeningeal metastasis, Epidermal growth factor receptor, Furmonertinib, Intrathecal Pemetrexed

## Abstract

**背景与目的:**

软脑膜转移（leptomeningeal metastasis, LM）是表皮生长因子受体（epidermal growth factor receptor, *EGFR*）突变型非小细胞肺癌（non-small cell lung cancer, NSCLC）的严重并发症，预后极差。第三代EGFR-酪氨酸激酶抑制剂（EGFR-tyrosine kinase inhibitors, EGFR-TKIs）增加剂量虽可提升中枢神经系统药物浓度，但其单药疗效仍不足以有效控制疾病进展。培美曲塞鞘内注射（intrathecal Pemetrexed, IP）能够绕过血脑屏障，直接作用于脑脊液，为LM的局部治疗提供了新途径。然而，目前关于高剂量第三代EGFR-TKIs伏美替尼（160 mg/d）联合IP治疗*EGFR*突变型NSCLC-LM的临床疗效与安全性的研究数据仍较缺乏。因此，本研究旨在评估该联合方案在此类难治性患者中的有效性及安全性，以期为临床实践提供真实世界证据。

**方法:**

选择2021年6月至2024年12月南京大学医学院附属鼓楼医院收治的40例*EGFR*突变型NSCLC-LM患者，其治疗方案均为高剂量伏美替尼（160 mg/d）联合IP治疗。通过系统收集患者的临床病例资料及随访信息，分析其颅内无进展生存期（intracranial progression-free survival, iPFS）和总生存期（overall survival, OS）、总体反应率和不良事件。生存分析采用*Kaplan-Meier*法，预后影响因素通过*Cox*比例风险回归进行多变量分析。

**结果:**

中位随访时间20.0个月，高剂量伏美替尼联合IP治疗在*EGFR*突变型NSCLC-LM患者中展现出显著疗效：总体反应率为85.0%，iPFS为9.6个月，中位OS为12.6个月，6、12和24个月的OS率分别为79.9%、53.9%和27.0%。多因素分析表明，联合贝伐珠单抗治疗是独立的保护性预后因素[风险比（hazard ratio, HR）=0.283, 95%CI: 0.114-0.702, *P*=0.006]，而基线卡氏体能状态（Karnofsky performance status, KPS）评分较差是独立危险因素（HR=3.069, 95%CI: 1.313-7.170, *P*=0.010）。安全性方面，主要不良事件为骨髓抑制（42.5%）、转氨酶升高（22.5%）和消化道反应（恶心和/或呕吐20.0%），多数为1-2级。仅报告1例4级骨髓抑制，经支持治疗后好转。

**结论:**

高剂量伏美替尼联合IP在*EGFR*突变型NSCLC-LM患者中表现出显著疗效和可控的安全性，联合贝伐珠单抗可能进一步增加临床获益，为难治性患者提供了有前景的治疗策略。

## Abstract (en)

**Background and objective:**

Leptomeningeal metastasis (LM) is a devastating complication of epidermal growth factor receptor (*EGFR*)-mutated non-small cell lung cancer (NSCLC), with a poor prognosis. While high-dose third-generation EGFR-tyrosine kinase inhibitors (EGFR-TKIs) can enhance drug concentrations in the central nervous system, their efficacy as monotherapy remains limited. Intrathecal Pemetrexed (IP) offers a promising local treatment approach by bypassing the blood-brain barrier and acting directly within the cerebrospinal fluid. However, clinical data on the efficacy and safety of combining high-dose third-generation EGFR-TKIs Furmonertinib (160 mg/d) with IP in *EGFR*-mutant NSCLC-LM patients are still scarce. Therefore, this study aims to evaluate the efficacy and safety of this combination regimen in this population to provide real-world data support for clinical practice.

**Methods:**

In this retrospective study, 40 patients with *EGFR*-mutant NSCLC-LM were enrolled at Nanjing Drum Tower Hospital, Affiliated Hospital of Medical School, Nanjing University between June 2021 and December 2024. All patients received oral Furmonertinib (160 mg/d) combined with IP. Primary endpoints were intracranial progression-free survival (iPFS), overall survival (OS), overall response rate (ORR) and adverse events. Survival analysis was performed using the *Kaplan-Meier* method, and prognostic factors were assessed via *Cox* proportional hazards regression by collecting the clinical data and follow-up information of the patients.

**Results:**

After a median follow-up of 20.0 months, the combination therapy demonstrated significant efficacy. The ORR was 85.0%, with a median iPFS of 9.6 months and a median OS of 12.6 months. The 6-, 12- and 24-month OS rates were 79.9%, 53.9% and 27.0%, respectively. Multivariate analysis identified combination therapy with Bevacizumab as an independent protective factor [hazard ratio (HR)=0.283, 95%CI: 0.114-0.702, *P*=0.006], while a poor baseline Karnofsky performance status (KPS) score was an independent risk factor (HR=3.069, 95%CI: 1.313-7.170, *P*=0.010). Adverse events were primarily grade 1-2, including myelosuppression (42.5%), elevated transaminases (22.5%), and gastrointestinal reactions (nausea and/or vomiting, 20.0%). Only one case of grade 4 myelosuppression was reported and resolved after supportive care.

**Conclusion:**

High-dose Furmonertinib combined with IP is an effective and well-tolerated regimen for *EGFR*-mutant NSCLC-LM. The addition of Bevacizumab may further improve outcomes, offering a promising strategy for refractory patients.

非小细胞肺癌（non-small cell lung cancer, NSCLC）进展过程中，有3%-5%的患者会发生软脑膜转移（leptomeningeal metastasis, LM），即肿瘤细胞播散至脑脊液腔，进而导致脑脊液循环障碍和神经系统功能损害^[1]^。LM的临床表现复杂多样，包括颅内压增高症状（如头痛、恶心、呕吐）、癫痫发作、意识障碍以及颅神经受累等多种神经系统异常。值得注意的是，由于靶向治疗带来的生存获益以及肿瘤自身的生物学特性差异，表皮生长因子受体（epidermal growth factor receptor, *EGFR*）突变阳性的NSCLC患者发生LM的风险更高，可达9.4%^[2]^。随着影像学技术和治疗手段的不断进步，LM的检出率和发生率呈持续上升趋势。然而，LM患者预后极差，未经治疗的患者中位总生存期（overall survival, OS）通常不足3个月^[1]^。因此，LM的临床管理仍是当前面临的重大挑战，亟需探索更有效的治疗策略。

EGFR-酪氨酸激酶抑制剂（EGFR-tyrosine kinase inhibitors, EGFR-TKIs）是晚期*EGFR*突变型NSCLC患者的标准一线治疗方案。然而，LM的出现仍是治疗过程中的严峻挑战，患者预后极差。此类患者治疗选择有限：全身化疗因血脑屏障的存在，药物在脑脊液中难以达到有效浓度，疗效受限；全脑放疗（whole brain radiation therapy, WBRT）虽可用于姑息缓解，但对弥漫性LM病灶的控制效果并不理想^[3,4]^。尽管第一、二代EGFR-TKIs对颅外病灶具有显著效果，但其中枢神经系统（central nervous system, CNS）穿透性较弱，在LM患者中的疗效仍较有限，中位OS仅约5.3个月^[5]^。随着第三代EGFR-TKIs的研发与应用，其血脑屏障穿透能力更强，在合并脑膜或脑实质转移的*EGFR*突变型NSCLC中显示出更好的治疗潜力，为LM患者的治疗提供了新的方向^[6]^。

伏美替尼作为一种新型国产第三代EGFR-TKIs，具有良好的CNS穿透性和广谱*EGFR*突变覆盖范围。临床研究^[7]^提示，伏美替尼在耐药后仍具备一定的疗效，且在高剂量应用时，其在脑脊液中的药物浓度显著提升，为LM的治疗提供了新思路。另一方面，鞘内注射化疗可绕过血脑屏障，将药物直接递送至蛛网膜下腔，从而在局部达到有效治疗浓度，增强对脑膜病灶的杀伤作用。在鞘内化疗药物中，培美曲塞因其耐受性较好且神经毒性较低，近年来已被探索用于LM的治疗^[8,9]^。因此，探索高效、安全的联合治疗策略，特别是基于高剂量第三代EGFR-TKIs的治疗方案，对改善*EGFR*突变型NSCLC-LM患者的预后具有重要的临床意义。本研究旨在通过系统性回顾接受高剂量伏美替尼联合培美曲塞鞘内化疗（intrathecal Pemetrexed, IP）的*EGFR*突变型NSCLC-LM患者的临床资料，评估该联合方案的疗效与安全性，以期为该类难治性患者群体提供新的治疗策略，并为后续前瞻性临床研究提供依据。

## 1 资料与方法

### 1.1 研究对象

本项回顾性研究的病例资料来源于2021年6月至2024年12月在南京大学医学院附属鼓楼医院连续确诊并接受治疗的*EGFR*突变型NSCLC-LM患者。纳入标准：（1）组织病理确诊为NSCLC且基因检测证实携带*EGFR*突变；（2）确诊LM需满足以下任一标准：脑脊液细胞学检查呈阳性，或头颅磁共振成像（magnetic resonance imaging, MRI）呈现典型特征（如脑膜异常强化或脑积水等表现）；（3）接受高剂量伏美替尼（160 mg/d）联合IP治疗至少2个周期。为确保能客观评估本联合方案的疗效与安全性，本研究要求入组患者从末次全身化疗或免疫治疗结束至本研究方案开始，需经历至少4周的洗脱期；末次放疗结束至本研究方案开始，需间隔至少2周。排除标准：（1）合并其他恶性肿瘤；（2）既往接受培美曲塞鞘内治疗且不耐受；（3）严重心、肝、肾功能不全；（4）诊断、治疗记录不完整或失访。本研究获南京大学医学院附属鼓楼医院伦理委员会批准（批准号：2023-562-02）。

### 1.2 治疗方案

本研究中所有患者均接受高剂量伏美替尼（160 mg，每日1次）口服治疗，并联合IP治疗，治疗持续至疾病进展或出现不可耐受的毒性反应。

IP具体方案如下：培美曲塞通过腰椎穿刺或Ommaya囊进行鞘内注射，每次给药剂量为20-30 mg，每个治疗周期的给药日程设定为第1和8天，周期长度为4周。IP持续至颅内病灶进展，或患者产生无法耐受的不良反应时方可终止。为预防化学性蛛网膜炎等潜在不良反应，在每次注射培美曲塞前，均会常规预先鞘内注射5 mg地塞米松。同时，所有患者均需接受辅助用药，具体包括：每日1次口服400 μg叶酸，以及在每个治疗周期首次给药前1-2周肌肉注射1 mg维生素B12，并在后续周期中重复此操作。这些辅助用药旨在减轻培美曲塞的全身毒性，提高治疗耐受性。

在全程治疗期间，允许联合贝伐珠单抗（400 mg/周期）进行抗血管生成治疗，而禁止合并使用其他类型的静脉化疗药物、放射治疗或免疫检查点抑制剂等治疗。允许纳入在研究开始前已接受过上述相关治疗的LM患者。

### 1.3 疗效与安全性评估

本研究采用神经肿瘤学软脑膜转移疗效评估标准（Response Assessment in Neuro-oncology Leptomeningeal Metastasis, RANO-LM）进行疗效评价^[10]^。该标准综合了神经系统检查、脑脊液细胞学及MRI评估。鉴于脑脊液细胞学检测可能存在假阴性结果，本研究对标准RANO-LM流程进行了针对性改良，主要包括：（1）临床症状评估：研究人员在每个治疗周期开始时，均对患者进行标准化的神经系统检查，并量化记录卡氏体能状态（Karnofsky performance status, KPS）评分与头痛程度。其具体判定标准为：“改善”定义为症状显著缓解或KPS评分提高≥20分；“稳定”指变化不明显；“进展”则为症状加重或KPS评分下降≥20分。（2）影像学评估：本研究中患者每2个治疗周期接受1次脑膜增强MRI检查，并由2位互不知情的影像学专家独立判读结果。影像学结果的判定严格遵循RANO-LM标准，最终结论分为缓解（包括完全缓解、部分缓解）、稳定或进展。（3）脑脊液生物学标志物动态监测：本研究在每个治疗周期通过腰椎穿刺或Ommaya囊采集脑脊液样本，并检测其癌胚抗原（carcinoembryonic antigen, CEA）水平。脑脊液CEA作为一项动态监测与参考指标，用于纵向追踪肿瘤负荷的变化趋势，为临床决策提供辅助信息，但它不作为独立的疗效判定标准。（4）最终综合疗效判定：最终疗效基于上述3个方面结果进行整合判断。疗效判定主要分为反应、稳定、进展。当各项结果不一致时，遵循“临床症状与影像学优先”的原则。脑脊液CEA的变化趋势用于辅助印证临床判断，但不推翻由核心依据得出的结论。疗效的统计汇总采用的指标包括：①总体反应率：在最终疗效判断中，达到“反应”的患者例数占总分析人群的百分比。②疾病控制率：达到“反应”与“稳定”的患者例数之和，占总分析人群的百分比。

生存分析中，颅内无进展生存期（intracranial progression-free survival, iPFS）定义为从启动高剂量伏美替尼联合IP治疗方案之日起，至首次出现颅内疾病进展或任何原因导致死亡的时间点。OS的计算则从联合治疗开始，截止至末次随访日（2025年6月30日）或因任何原因死亡的时间。不良反应主要包括骨髓抑制、肝功能异常、皮疹、消化道反应及间质性肺病。所有不良事件的严重程度均参照《不良事件通用术语标准5.0版》进行定义和分级。

### 1.4 统计学方法

本研究所有数据分析均使用SPSS 27.0统计学软件完成。生存分析采用*Kaplan-Meier*法绘制生存曲线，组间比较采用*Log-rank*检验。为识别影响预后的独立因素，首先采用单因素*Cox*比例风险回归模型对潜在有关变量进行初步筛选，随后将单因素分析中*P*<0.10的变量纳入多因素*Cox*比例风险回归模型进一步分析。所有统计学检验均为双侧检验，*P*<0.05为差异有统计学意义。

## 2 结果

### 2.1 临床特征

根据纳入标准和排除标准，最终本研究共纳入40例*EGFR*突变型NSCLC-LM患者。具体筛选流程图见图1。40例LM患者基线临床特征如表1所示。所有患者中位年龄为56（范围：34-72）岁，男性23例（57.5%），女性17例（42.5%）。功能状态评估显示，KPS评分≥60分与<60分者各占50.0%。基因突变谱以19外显子缺失突变（*n*=19, 47.5%）和21外显子L858R点突变（*n*=16, 40.0%）为主，其余为少见突变类型，包括L861Q、G719X、T790M突变各1例（2.5%）、2例（5.0%）、1例（2.5%），另有1例（2.5%）为L858R合并20外显子突变复合型。诊断LM时，62.5%（25/40）的患者合并脑实质转移，80.0%（32/40）伴有颅外转移灶。LM确诊依据方面，脑脊液细胞学阳性率为92.5%（37/40），MRI检查阳性率为77.5%（31/40）。既往治疗史显示，42.5%（17/40）的患者曾接受至少三线系统治疗；20.0%（8/40）在LM诊断后接受过静脉化疗，中位周期数为4（范围：2-6）个。本研究期间，55.0%（22/40）的患者联合使用了贝伐珠单抗（400 mg/周期）静脉治疗，中位用药6（范围：2-12）个周期；15.0%（6/40）曾接受颅脑放疗，均为合并脑实质转移者。鞘内给药途径以腰椎穿刺为主（77.5%, 31/40），Ommaya囊植入给药占22.5%（9/40）；37.5%（15/40）的患者完成了不少于10次鞘内治疗。

**图 1:**
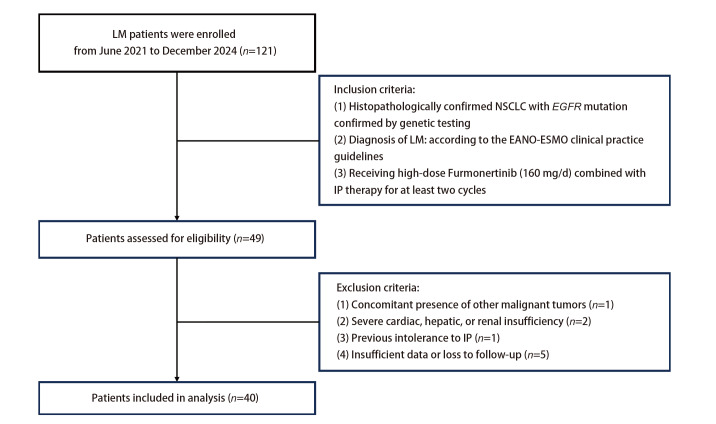
研究筛选流程图

**表 1:** 患者的基线特征（*n*=40）

Variables	*n* (%)
Age (yrs)	
≥60	11 (27.5)
<60	29 (72.5)
Gender	
Male	23 (57.5)
Female	17 (42.5)
KPS score	
≥60	20 (50.0)
<60	20 (50.0)
EGFR mutations	
Exon 19 deletion	19 (47.5)
Exon 21 L858R	16 (40.0)
Exon 18 G719A/G719S	2 (5.0)
Exon 21 L861Q	1 (2.5)
Exon 20 T790M	1 (2.5)
Compound mutation	1 (2.5)
Brain metastases	
Yes	25 (62.5)
No	15 (37.5)
Extracerebral metastases	
Yes	32 (80.0)
No	8 (20.0)
CSF cytology	
Positive	37 (92.5)
Negative	3 (7.5)
MRI	
Positive	31 (77.5)
Negative	9 (22.5)
Furmonertinib treatment lines	
<3	23 (57.5)
≥3	17 (42.5)
Systemic chemotherapy after LM	
Yes	8 (20.0)
No	32 (80.0)
Brain radiotherapy after LM	
Yes	6 (15.0)
No	34 (85.0)
Bevacizumab after LM	
Yes	22 (55.0)
No	18 (45.0)
Intrathecal administration	
Ommaya reservoir	9 (22.5)
Lumbar puncture	31 (77.5)
IP number	
≥10	15 (37.5)
<10	25 (62.5)

KPS: Karnofsky performance status; CSF: cerebrospinal fluid; MRI: magnetic resonance imaging

本研究纳入的40例LM患者中，神经系统临床表现多样，其中以颅内压增高相关症状最为常见。具体而言，头痛者24例（60.0%），恶心和/或呕吐者16例（40.0%）。头晕亦是常见症状，占32.5%（*n*=13）。此外，观察到局灶性神经功能缺损表现，包括视神经受累6例（15.0%），癫痫发作或抽搐4例（10.0%）；部分患者亦出现听力下降、步态不稳、认知障碍及咀嚼肌无力等症状（表2）。

**表 2:** 患者临床症状（*n*=40）

Clinical manifestation	*n* (%)
Headache	24 (60.0)
Nausea and/or vomiting	16 (40.0)
Dizziness	13 (32.5)
Optic nerve involvement	6 (15.0)
Auditory nerve involvement	2 (5.0)
Epilepsy or convulsion	4 (10.0)
Walking instability	4 (10.0)
Cognitive impairment	2 (5.0)
Masticatory muscle dysfunction	1 (2.5)

### 2.2 疗效评估

#### 2.2.1 临床有效性评估

本研究中，多数患者经高剂量伏美替尼联合IP治疗后，表现出显著的临床获益。如表3所示，85.0%的患者神经系统症状（包括头痛、恶心、呕吐等）获得明显缓解。同时，患者的功能状态得到改善，42.5%的患者KPS评分较前提升。在25例基线脑脊液CEA升高的LM患者中，88.0%（*n*=22）的患者CEA水平较治疗前降低。影像学评估表明，22.5%的患者脑膜强化灶缩小，65.0%的患者病灶维持稳定。综合症状、影像及脑脊液指标评估，本研究方案的总体反应率为85.0%，疾病控制率达92.5%，证实该联合策略对颅内病灶具有良好的控制效果。图2通过展示代表性患者治疗前后的脑膜病灶对比，直观反映了这一治疗效果。

**表 3:** 临床疗效评估

Items	*n/N* (%)
Clinical symptom assessment	
Neurological symptom remission	34/40 (85.0)
KPS elevation	17/40 (42.5)
Headache improved	21/24 (87.5)
Radiologic evaluation	
Improved	9/40 (22.5)
Stable	26/40 (65.0)
Worse	3/40 (7.5)
Not review	2/40 (5.0)
CSF CEA reduction	22/25 (88.0)
Overall response assessment	
Response	34/40 (85.0)
Stable	3/40 (7.5)
Progression	3/40 (7.5)

CEA: carcinoembryonic antigen.

**图 2:**
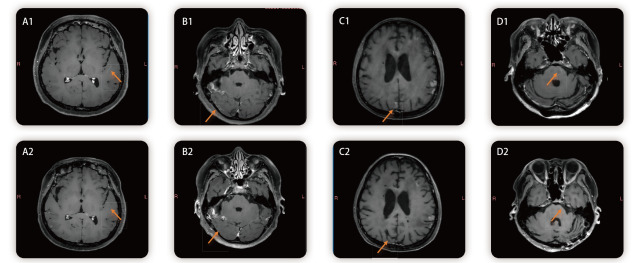
LM患者治疗前后脑膜病灶的MRI图像对比。A-D为不同患者的MRI对比，1为治疗前，2为治疗后。橙色箭头指示脑膜病灶区域，可见多数患者治疗后强化信号减退、病灶缩小，提示脑膜病变明显改善。

#### 2.2.2 生存分析

截至2025年6月30日，中位随访时间为20.0个月。至随访结束时，共记录到26例（65.0%）患者死亡，14例（35.0%）患者存活。生存分析结果揭示，本队列中LM患者的中位iPFS为9.6个月，中位OS为12.6个月（图3）。对不同随访时间点的生存率进行估算，得出6、12及24个月的OS率分别为79.9%、53.9%和27.0%。亚组分析进一步明确，基线KPS评分是显著的预后影响因素：评分≥60分的患者中位OS达到15.9个月，显著优于评分<60分患者的10.5个月（*P*=0.0034）。此外，治疗过程中联合贝伐珠单抗亦展现出明确的生存优势，联合治疗者的中位OS为18.3个月，而未联合治疗者为11.7个月，差异具有统计学意义（*P*=0.0019）（图4）。多因素*Cox*回归分析进一步确认，KPS评分较差是预后不良的独立危险因素[风险比（hazard ratio, HR）=3.069, 95%CI: 1.313-7.170, *P*=0.010]。而联合贝伐珠单抗治疗是预后的独立保护因素（HR=0.283, 95%CI: 0.114-0.702, *P*=0.006）（表4）。其他因素如性别、年龄、*EGFR*突变类型以及是否伴有脑转移或颅外转移等，在本研究中未显示出与预后存在显著关联。

**图 3:**
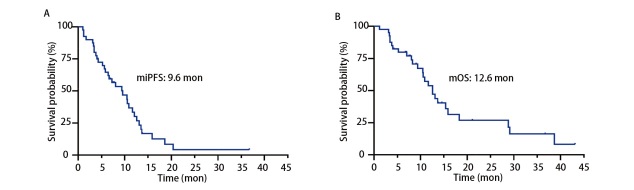
40例NSCLC-LM患者的iPFS（A）和OS（B）曲线

**图 4:**
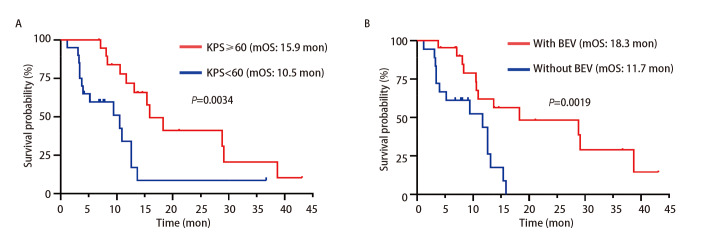
不同亚组患者OS的*Kaplan-Meier*曲线。A：KPS≥60分和 KPS<60分组的生存曲线；B：BEV组和未使用BEV组的生存曲线。

**表 4:** LM患者总生存期的单因素和多因素*Cox*回归分析

Variable	Univariate analysis			Multivariate analysis	
	HR (95%CI)	P		HR (95%CI)	P
Age (≥60 *vs* <60 yrs)	0.889 (0.371-2.129)	0.792			
Gender (Male vs Female)	0.963 (0.425-2.184)	0.929			
KPS score (<60 *vs* ≥60)	3.281 (1.434-7.507)	0.005		3.069 (1.313-7.170)	0.010
Gene mutation (Uncommon *vs* Common)	1.042 (0.242-4.491)	0.956			
MRI (Positive *vs* Negative)	0.896 (0.414-1.941)	0.773			
Brain metastases (Yes *vs* No)	1.119 (0.498-2.516)	0.785			
Extra-CNS metastases (Yes *vs* No)	1.473 (0.579-3.748)	0.417			
Combined with BEV (Yes *vs* No)	0.265 (0.109-0.646)	0.004		0.283 (0.114-0.702)	0.006
Radiotherapy for CNS after LM (Yes *vs* No)	0.968 (0.223-4.206)	0.965			
Chemotherapy after LM (Yes *vs* No)	0.747 (0.279-1.999)	0.561			
Intrathecal administration (Ommaya reservoir *vs* Lumbar puncture)	0.863 (0.320-2.327)	0.772			

CNS: central nervous system.

### 2.3 安全性评估

本治疗方案整体耐受性良好，未发生治疗相关严重不良事件。大多数不良反应为1-2级，主要包括骨髓抑制（42.5%）、转氨酶升高（22.5%）以及恶心和/或呕吐（20.0%）。部分患者合并出现贫血、白细胞减少或血小板减少等血液学指标异常。仅1例患者出现4级骨髓抑制，经支持治疗后完全恢复，无永久性器官损伤。值得注意的是，治疗过程中未观察到鞘内化疗相关的神经毒性或药物性间质性肺炎。与伏美替尼相关的皮疹（5.0%）和腹泻（7.5%）发生率较低且程度轻微，未导致治疗中断。不良反应数据见表5。

**表 5:** 不良反应[*n* (%)]

Toxicity	Any grade	Grade 1	Grade 2	Grade 3	Grade 4
Myelosuppression	17 (42.5)	5 (12.5)	8 (20.0)	3 (7.5)	1 (2.5)
Anaemia	6 (15.0)	3 (7.5)	1 (2.5)	2 (5.0)	0 (0.0)
Leukopenia	13 (32.5)	3 (7.5)	7 (17.5)	2 (5.0)	1 (2.5)
Thrombocytopenia	3 (7.5)	1 (2.5)	2 (5.0)	0 (0.0)	0 (0.0)
Elevated ALT/AST	9 (22.5)	7 (17.5)	2 (5.0)	0 (0.0)	0 (0.0)
Rash	2 (5.0)	2 (5.0)	0 (0.0)	0 (0.0)	0 (0.0)
Nausea and/or vomiting	8 (20.0)	7 (17.5)	1 (2.5)	0 (0.0)	0 (0.0)
Diarrhea	3 (7.5)	1 (2.5)	2 (5.0)	0 (0.0)	0 (0.0)
Elevated γ-GT	8 (20.0)	6 (15.0)	1 (2.5)	1 (2.5)	0 (0.0)
ILD	0 (0.0)	0 (0.0)	0 (0.0)	0 (0.0)	0 (0.0)

ALT: alanine aminotransferase; AST: aspartate aminotransferase; γ-GT: γ-glutamyl transpeptidase; ILD: interstitial lung disease.

## 3 讨论

本研究作为首项探讨高剂量伏美替尼（160 mg/d）联合IP治疗在*EGFR*突变型NSCLC-LM中疗效的回顾性研究，结果显示该联合方案可带来显著临床获益。40例NSCLC-LM患者的iPFS和OS分别可达9.6和12.6个月，总体反应率为85.0%，且安全性整体可控。该结果提示，高剂量伏美替尼联合IP治疗在改善*EGFR*突变型NSCLC-LM患者生存结局方面具有潜在临床应用价值，也为后续前瞻性研究提供了真实世界数据支持。

高剂量第三代EGFR-TKIs通过提升脑脊液中的药物浓度，已成为改善NSCLC-LM患者预后的重要策略。其疗效优势主要源于该类药物固有的高血脑屏障穿透能力，确保其能更有效地分布至CNS。BLOOM研究^[11]^显示，采用双倍剂量奥希替尼（160 mg/d）治疗*EGFR*突变型NSCLC-LM，其中位PFS为8.6个月，中位OS可达11.0个月，且不良反应可控。另有回顾性研究^[12]^表明，与标准剂量相比，高剂量第三代EGFR-TKIs可显著提升患者的临床缓解率（65.2% *vs* 45.8%, *P*=0.047）与中位OS（15.0 *vs* 10.2个月，*P*=0.014）。新近发表的一项回顾性真实世界研究^[13]^显示，在接受高剂量第三代EGFR-TKIs治疗的LM患者中，其中位OS显著优于接受标准剂量治疗的患者（19.7 *vs* 9.0个月）。这些数据共同表明，增加EGFR-TKIs剂量以提升颅内暴露浓度，是治疗LM的有效策略。

伏美替尼作为我国自主研发的第三代EGFR-TKIs，不仅已获批用于*EGFR*敏感突变晚期NSCLC患者的一线治疗，更因其卓越的CNS穿透性而受到关注。其活性代谢产物AST5902不仅与原型药物具有相似的抗肿瘤活性，且二者均具有较强亲脂性，不易被P-糖蛋白外排，从而能够更高效地穿透血脑屏障，在脑组织和脑脊液中达到较高的药物浓度^[14]^。临床前药代动力学研究^[15]^显示，伏美替尼在小鼠模型中表现出较奥希替尼更高的脑/血浆浓度比，且其疗效呈现剂量依赖性特征。多项临床研究^[16-18]^也证实，提高伏美替尼剂量可进一步提升颅内疗效。两项II期研究的汇总分析^[16]^显示，在52例伴CNS转移的*EGFR* T790M突变NSCLC患者中，接受160 mg/d伏美替尼治疗者的CNS-客观缓解率（objective response rate, ORR）为85%，CNS-PFS为19.3个月，均优于80 mg/d组（CNS-ORR: 65%；CNS-PFS: 11.6个月）。王启鸣教授团队^[17]^开展的一项前瞻性真实世界研究则探索了240 mg/d伏美替尼在*EGFR*突变型NSCLC-LM患者（包括多线治疗耐药、基线状态评分较差的难治人群）中的疗效，结果显示中位OS为8.43个月，其中第三代EGFR-TKIs未耐药患者的中位OS达到14.53个月，且安全性良好。这些证据均支持高剂量伏美替尼在控制颅内转移方面的潜力，并与本研究中观察到的生存获益趋势一致。

在联合治疗策略中，高剂量伏美替尼与IP的协同作用有望实现对LM的多维度控制。鞘内化疗可绕过血脑屏障，使化疗药物在蛛网膜下腔达到局部高浓度，直接作用于脑膜病灶。与传统鞘内化疗药物相比，培美曲塞因其多靶点抑制叶酸代谢特性、良好的耐受性及较低的神经毒性，已成为鞘内给药的重要选择^[1,19]^。研究^[19]^表明，IP治疗在*EGFR*突变型NSCLC-LM患者中表现出显著疗效，中位OS可达12.0个月，总体有效率为80.3%，且安全性良好。高剂量伏美替尼则通过提升脑脊液药物浓度，增强对全身及颅内病灶的系统控制。其协同作用在临床实践中得到验证。一项纳入23例EGFR-TKIs耐药后LM患者的回顾性研究^[20]^也证实了这一联合策略的潜力，该研究中患者均接受规律IP治疗联合高剂量第三代EGFR-TKIs（奥希替尼160 mg/d、伏美替尼160 mg/d或阿美替尼165 mg/d），结果显示颅内疾病控制率达86.96%，中位PFS为10个月，中位OS为12个月。该结果验证了“全身靶向治疗联合局部鞘内化疗”这一双途径策略在难治性LM治疗中的临床应用潜力，为这类患者提供了新的治疗方向。未来仍需更大样本的临床研究及真实世界数据进一步验证其长期疗效与适用范围。

在LM的鞘内治疗中，给药途径的选择与药物剂量的确定是影响疗效与安全性的关键环节。目前临床主要采用经Ommaya囊给药与腰椎穿刺给药两种方式。Ommaya囊作为一种植入式储液装置，可将药物直接注入脑室，有利于药物随脑脊液循环在蛛网膜下腔均匀分布，避免重复腰穿所致的不便与并发症，尤其适合需长期多次鞘内给药的患者。然而，该方式需手术植入，存在一定感染、导管堵塞或出血等风险。腰椎穿刺为传统鞘内给药方式，操作简便、创伤小且成本较低，但对长期多次给药不如Ommaya囊便利，且术后可能发生低颅压性头痛、局部血肿或感染等并发症^[21]^。在本研究的探索性分析中，我们观察到通过腰椎穿刺与Ommaya囊给药的两种途径相比较，患者的OS未见统计学显著差异。由于本研究样本量有限，此结果尚不足以得出“两种方式等效”的确切结论，但可能提示在规范操作与护理下，两种途径均具备可行性。这一发现与既往一项回顾性研究^[19]^的结果方向一致 。该结果的稳健性及普遍适用性，有待未来在前瞻性、大样本研究中进一步验证。目前，IP的最佳剂量与给药频率尚未形成统一标准，各类临床研究中探索的剂量范围较广，通常为10-50 mg/次^[19,22]^。本团队前期开展的剂量爬坡试验表明，培美曲塞单次鞘内给药剂量在20-30 mg，患者表现出良好的耐受性。基于此前期经验，并结合本研究所有患者均同步接受高剂量伏美替尼（160 mg/d）全身治疗的背景，为在保障疗效的同时最大限度控制潜在的叠加毒性，本研究最终选择将培美曲塞的每次鞘内给药剂量设定为20-30 mg。临床观察结果显示，在此剂量方案下，患者治疗反应良好，且未出现不可控的药物相关性毒性反应。这一剂量范围为后续研究提供了重要参考，但其最佳给药方案仍有待更大样本的前瞻性研究进一步确认。

本研究的单因素与多因素分析结果共同提示，KPS评分与联合贝伐珠单抗治疗是LM患者生存预后的独立影响因素。这不仅具有预后判断价值，更具重要的预测意义，为临床筛选可能从本联合方案中获得长期获益的优势人群提供了线索。KPS评分作为一项广泛应用的量化工具，能有效反映肿瘤患者的全身功能状态和治疗耐受能力。评分较高的患者通常具有更佳的身体机能储备和免疫状态，从而对高剂量EGFR-TKIs联合鞘内化疗等强度较大的综合治疗方案具备更好的耐受性。此外，高KPS评分患者往往治疗依从性更优，能够完成更多周期的治疗，进而有望获得更持久的疾病控制。吴一龙教授团队^[23]^也证实KPS评分是肺癌脑膜转移的关键预后指标，并建立预后模型，对患者风险分层具有重要价值。本研究还发现，接受贝伐珠单抗联合治疗组的LM患者OS显著延长（18.3 *vs* 11.7个月，*P*=0.0019）。从作用机制来看，贝伐珠单抗作为一种抗血管内皮生长因子（vascular endothelial growth factor, VEGF）的单克隆抗体，可通过特异性结合VEGF，阻断其与受体相互作用，从而抑制肿瘤组织内未成熟血管的生成，并促进残存血管的结构和功能正常化。这一“血管正常化”效应有助于改善肿瘤微循环灌注效率，提高系统性治疗药物（如EGFR-TKIs或化疗药物）在肿瘤组织尤其是血脑屏障内的递送浓度与分布，从而增强抗肿瘤效果。此外，抑制病理性血管生成本身也可直接遏制肿瘤的生长和远处播散。多项临床研究^[18,24]^已证实贝伐珠单抗在NSCLC-LM治疗中的协同价值。一项回顾性分析^[24]^表明，奥希替尼联合贝伐珠单抗可能通过调节E-钙黏蛋白表达并增加奥希替尼在脑组织中的暴露量，产生协同抗肿瘤效应，使得联合治疗组患者的中位OS较奥希替尼单药组显著延长（18.0 *vs* 13.7个月，*P*=0.046）。Pan等^[18]^开展的一项研究纳入78例第三代EGFR-TKIs耐药后伴脑转移的NSCLC患者，结果显示高剂量伏美替尼（160 mg/d）联合贝伐珠单抗治疗后，颅内客观缓解率为37.1%，中位iPFS为7.2个月；在合并LM的患者亚组中，iPFS进一步延长至7.63个月，提示该方案对CNS转移灶具有持续控制能力。这些研究共同提示，贝伐珠单抗可能通过改善脑膜病灶周围血管功能与药物渗透性，与靶向治疗或化疗形成协同效应。对于条件允许的患者，积极联合贝伐珠单抗可能是一种有望提升长期疗效的策略。但高剂量伏美替尼、鞘内化疗与贝伐珠单抗三联疗法的有效性与安全性仍需更大样本的前瞻性研究进一步验证。

本研究观察到的主要不良事件为骨髓抑制与转氨酶升高，多数为1-2级。仅1例患者出现4级骨髓抑制，经支持治疗后恢复，未发现治疗相关的显著鞘内神经毒性或间质性肺炎。该安全性与近期鞘内培美曲塞化疗研究结果一致，表明IP总体耐受性良好，其风险主要集中在骨髓抑制、肝功能异常及局部穿刺相关并发症^[19,22]^。高剂量伏美替尼可能引起皮疹、腹泻及心脏毒性等反应，需在治疗中密切监测^[18,20]^。因此，联合治疗时应加强对血常规、肝肾功能、心电图及肺功能的定期随访，并配合积极支持治疗，以保障用药安全。

本研究虽取得积极结果，但仍存在一些局限。作为一项单中心回顾性研究，其样本量相对有限，且研究设计本身难以完全避免选择偏倚和信息偏倚的存在。小样本量也可能限制多因素分析时对某些潜在混杂因素（如联合治疗方案）的统计效能。其次，在临床实践中，难以排除其他联合治疗方案（如放疗、系统性化疗或免疫治疗）带来的混杂效应。此外，本研究引入脑脊液CEA水平作为动态监测疗效的参考指标，其敏感性与特异性存在固有局限；未来研究可探索整合脑脊液循环肿瘤细胞（circulating tumor cells, CTCs）等更具特异性的新型液体活检技术，以期实现更精准的疗效监控与预后判断。另外，本研究缺乏随机对照设计，疗效评价主要依赖于历史研究数据的外部对照。未来仍需通过大样本前瞻性、多中心、随机化临床试验来进一步验证本研究的初步结论。

本研究表明，对于*EGFR*突变型NSCLC-LM患者，高剂量伏美替尼联合IP治疗方案具有显著的疗效和良好的安全性。联合贝伐珠单抗被证实为积极的独立预后因素，进一步提示其在整体治疗策略中可能增强生存获益。本研究结果支持该联合方案在此类难治人群中的临床应用潜力，未来研究方向可聚焦于在前瞻性设计中验证其疗效，并探索该方案用于早期治疗阶段的可行性，以期进一步提升患者长期预后。
